# Supplementary material for: Development of chemokine network inhibitors using combinatorial saturation mutagenesis
Source: Commun Biol. 2025 Apr 3;8:549. doi: 10.1038/s42003-025-07778-6 (PMC11969024; doi:10.1038/s42003-025-07778-6)
Supplement: Supplementary file 4 — Supplementary Data 2 [file 42003_2025_7778_MOESM4_ESM.zip › SupplementaryData3/scripts/xattune_ic50.tar!/./script/analysis/JK/JK_R37_E76_INS_HD2CM304_ATC_plot.pdf]

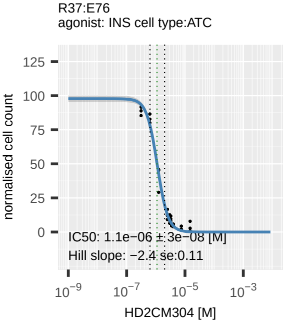

R37:E76
agonist: INS cell type:ATC
normalised cell count
125
100
75
50
25
0
IC50: 1.1e−06 ± 3e−08 [M]
Hill slope: −2.4 se:0.11
10^{-9}
10^{-7}
10^{-5}
10^{-3}
HD2CM304 [M]
